# Quality of care for type 2 diabetes in Jordan: A national study

**DOI:** 10.3892/mi.2023.87

**Published:** 2023-05-26

**Authors:** Rami Saadeh, Haya Alsmadi, Anwar Batieha, Yousef Khader, Hashem Jaddou, Mohammed El-Khateeb, Mohammed Z. Allouh, Kamel Ajlouni

**Affiliations:** 1 Department of Public Health and Community Medicine, College of Medicine, Jordan University of Science and Technology, Irbid 22110, Jordan; 2 Jerash Medical Center, Dental Department, Jerash Governorate, Jordan Ministry of Health, Amman 11118, Jordan; 3 The National Center (Institute) for Diabetes, Endocrinology and Genetics (NCDEG), University of Jordan, Amman 11942, Jordan; 4 Department of Anatomy, Faculty of Medicine, Jordan University of Science and Technology, Irbid 22110, Jordan

**Keywords:** type 2 diabetes, quality of care, management, glycemic control, healthcare provider

## Abstract

The present study aimed to describe the quality of healthcare delivered to patients with type 2 diabetes in Jordan in 2017. Another objective was to identify the factors related to glycemic control and hospital admission due to type 2 diabetes. This was a national population-based household study. Aspects of care quality were evaluated in relation to outcomes, such as glycemic control [hemoglobin A1c; glycated hemoglobin (HbA1c) level <7%] and hospital admission owing to diabetes. A total of 754 patients previously diagnosed with type 2 diabetes and aged ≥25 years were recruited. The number of annual visits was >10 for 48.5% and 1-4 for 38.2% of patients. The proportion of patients achieving glycemic control was 33.0%. In total, 4 of 5 patients reported easy access to health facilities and good health team support. Foot and eye examinations were performed for 24.9 and 55.0% of the patients, respectively. Dietary advice was delivered to 87.5% of the patients. Glycemic control exhibited a significant inverse association with the duration of diabetes and the number of annual visits. Following a specific diet for managing diabetes and the cessation of medication after an improved well-being were independently associated with a higher likelihood of glycemic control (HbA1c <7%). On the whole, the present study demonstrates that a number of indicators for the quality of diabetes care in Jordan were relatively satisfactory; however, others require improvement. The findings demonstrate that numerous patients with diabetes in Jordan require education about the treatment and management of, and complications associated with diabetes, especially those who are recently diagnosed.

## Introduction

Healthcare services play a major role in dealing with the increasing burden of diabetes mellitus; however, the elements of ideal healthcare services for diabetes have not yet been well defined. Enhancing knowledge about the most efficient aspects of diabetes care is critical and would aid in formulating health policies and care recommendations that best contribute to diabetes mellitus management. Globally, diabetes is considered a major public health concern and has adverse health and economic impacts (1). Several healthcare organizations are working towards developing the most cost-effective care measures to achieve the best health outcomes with minimal possible expenditures (2).

The prevalence of diabetes in Jordan has exhibited an increasing trend over the past three decades and its prevalence is one of the highest, not only regionally but also globally, which reached 23.7% in 2017 (3,4). The high prevalence and limited resources in Jordan warrant the provision of high-quality services. However, the initial and most essential step in improving healthcare services delivered to patients with diabetes is to determine the status of the provided care, which would assist in verifying the gap between the actual and optimal care quality and in highlighting the areas of weaknesses that need improvement. This would in turn help improve the health outcomes and quality of life of patients with diabetes through the efficient allocation of resources. Nevertheless, implementing any management care plan for diabetes should be successful at the following three levels: The health system, healthcare provider and patient (5).

In Jordan, there are limited data available on services provided to patients with diabetes and aspects of good quality of care (6). Evaluating the quality of diabetes healthcare services delivered in Jordan is a novel topic that has not yet been addressed in previous studies, at least to the best of our knowledge. Therefore, the present study was conducted to describe the health services provided to Jordanians with type 2 diabetes at a national level, to compare these services with the current standards of the American Diabetes Association (ADA), to estimate the proportion of patients with controlled diabetes and to investigate the association of this proportion with care quality measures employed. The findings of the present study may help to evaluate the performance of diabetes care programs in Jordan and provide the necessary information for the improvement. Given that the present study was a national and population-based study, the results may be of value to the field of diabetes care delivery.

## Patients and methods

The present study performed a secondary analysis of data that were collected by the collaboration of Ministry of Health, National Center for Diabetes, and Jordan University of Science and Technology, Irbid, Jordan.

### Study population

The study population was a national representation of the three regions (North, Middle and South) and all 12 governorates of Jordan. The first step was the selection of health centers in each governorate. The health director in each governorate had the responsibility to select one to three health centers that best represent the urban and rural residents in the directorate. The health centers need to have sufficient space for the study team, which was composed of 25 individuals and need to have the facilities to perform the required measurements and procedures. There are ~380 primary care centers in Jordan and a total of 17 primary health centers were randomly selected throughout the country. The study participants were selected on a household basis. Houses were elected systemically from the catchment areas of the selected health centers.

A team composed of a man and a woman visited the selected households and explained the purpose of the study. Household members (≥18 years of age) were invited to participate in the study. They were instructed to visit the health center the following day during the early morning hours (8:00 a.m.) and to fast for at least 10 h. The total number of participants was 4,056. The present study included only the participants with type 2 diabetes and an age ≥25 years.

### Response rate

The team encouraged individuals to participate in the study by working on holidays and weekends and offering free transportation. The response rate reached 94% of females and only 40% of males. The reason behind the wide discrepancy in the response rate between males and females was possibly due to the higher employment rate or the higher availability of women on these specific days of data collection.

### Data collection

The present study was multipurpose and a wide range of data were collected. Each participant was directly interviewed using a comprehensive structured questionnaire prepared for the purposes of the study. This questionnaire gathered sociodemographic data, data on cardiovascular risk factors, health care, lifestyle, dress style, mental health and other parameters. Measurements included height, weight, waist circumference, hip circumference and blood pressure. These measurements were obtained by well-trained staff of the health centers. Blood samples were collected by nurses or laboratory technicians for performing the intended laboratory tests. The samples were collected and centrifuged within 1 h. The centrifugation process lasted for 10 min, samples were then labeled, and were sent in ice boxes (under 10˚C) to the Central Laboratory of the National Centre of Diabetes, Endocrinology, and Genetics in Amman, Jordan. A well-trained group of laboratory technicians performed the required laboratory tests.

### Ethical concerns

Approvals were obtained from the Ethics Committee for Research on Humans of the National Center for Diabetes, Endocrinology, and Genetics in Amman, Jordan and from the Jordan University of Science and Technology Institutional Review Board (404-2022). A written letter was obtained from the Ministry of Health to ensure staff cooperation in the health centers. Information about all participants was treated confidentially and used only for research purposes. All participants signed consent forms and were given the right to withdraw their participation at any time without any penalty.

### Variable definitions

The following variables were used in the present study for the purposes of the analysis: i) Body mass index (BMI) was defined as the body mass in kilograms divided by the square of the body height in meters and is expressed in units of kg/m^2^. According to the American Heart Association (AHA), BMI was categorized as follows (AHA, 2023): Underweight, <18.5; normal weight, 18.5-24.9; overweight, 25-29.9; and obese, ≥30. ii) (7) Family history of diabetes: Classified as yes if at least one first-degree family member had type 2 diabetes. iii) Easy access to health facilities: Classified as yes if the patient stated that he/she could reach the treating physician easily. iv) Physical activity categories were based on the Physical Activity for Guidelines for Americans, 2nd edition (8) and was as follows: Light- intensity physical activity: Physical activities that do not lead to sweating and increased pulse rate. This variable was categorized according to the number of hours of exercise per week as follows: <1, 1-9, 10-19, and ≥20. Moderate-intensity physical activity: Everyday physical activities, including walking, cleaning, shopping, babysitting, cooking and garden work. This variable was categorized according to the number of hours of exercise per week as follows: <1, 1-9, 10-19 and ≥20. High-intensity physical activity: Physical activities, including brisk walking, digging in the ground, excavating soil, playing football, swimming and lifting weights. This variable was categorized according to the number of hours of exercise per week as follows: 0, 0.5-3 and >4. v) Health team support: This was based on the Likert scale: Very high, high, medium, low, very low and none. This variable was recorded into two levels: Good and poor. A good level of health team support comprised the responses very high, high, and medium, while the poor level indicated low, very low and none.

### Statistical analysis

The statistical analysis was conducted using the Statistical Package for Social Sciences software (SPSS version 22; IBM Corp.). Data cleaning was performed using the range and logical checks. Outliers were treated as missing data. The analysis involved sociodemographic variables, and provider- and patient-related aspects of health care. Each variable is expressed in a separate table with frequencies and percentages. The variable health team support was based on a Likert scale; very high, high, medium, low, very low and none. This variable was recorded into two levels: Good and poor. Good level of health team support comprised the responses very high, high and medium, while the poor level indicated low, very low and none. Bivariate analysis was performed to explore the associations between glycemic control [glycated hemoglobin (HbA1c) <7%] as the outcome variable and relevant variables as independent variables. The Chi-squared (χ^2^) test was used to assess the statistical significance of the observed differences. A P-value ≤0.05 was considered to indicate a statistically significant difference. Multivariate analysis using binary logistic regression was performed to identify the factors that were related to glycemic control (HbA1c <7%) after controlling for potential confounders, which included age, sex and physical activity. At the first step, all relevant variables, which exhibited a statistically significant difference or were close to it in the bivariate analysis, were included in the model. Subsequently, insignificant variables were excluded consecutively until the best fitting model with the least number of variables was reached that, best explained the variability in glycemic control. A similar approach was undertaken for the variable hospital admission due to diabetes during the past year as the outcome variable.

## Results

### Sociodemographic characteristics

The total number of participants recruited was 4,056, of whom 754 were aged ≥25 years and previously diagnosed with diabetes. Participants with newly discovered diabetes (n=161) were not eligible for inclusion in the present. The sociodemographic characteristics of the study population are presented in Table I. There was a higher proportion of females (57.1%) than males. The mean age of the study participants was 55.2±10.3 years. The majority of the participants were Jordanians and currently married. Approximately 66% of the participants did not receive >12 years of school education, while 11.2% were illiterate. Of the study participants, >90% were obese or overweight. Approximately 71% of the participants had at least one family member with diabetes.

The association between glycemic control (HbA1c level <7%) and the sociodemographic characteristics of the patients with diabetes is presented in Table I. Females were more likely to achieve glycemic control compared with males, and the difference was significant (P=0.003). The proportion of patients with controlled diabetes decreased as the age increased (P=0.002). The educational level was significantly related to glycemic control (HbA1c level <7%); the proportion of patients with controlled diabetes increased with an increase in the years of education received (P=0.007).

Provider-related aspects of healthcare quality. The characteristics of the study participants according to aspects of healthcare provision are presented in Table II. Approximately 54% of the patients received healthcare for diabetes in the facilities of the Ministry of Health. Insurance covers >85% of the patients with diabetes in Jordan. The number of annual follow-up visits was >10 for 48.5% and 1-4 visits for 38.2% of the patients, respectively, and 6.6% of the patients had not received medical care. In total, >81% of the patients reported that they could easily reach the health facility. Not all patients underwent a regular examination of their cholesterol and blood glucose levels, and blood pressure, or feet examination or eye examinations. However, the majority of patients reported a good team support and receiving advice regarding diet, going to the gym and losing weight.

The associations between glycemic control (HbA1c level <7%) and healthcare provider aspects are shown in Table II. Individuals with diabetes visiting the doctor >10 times/year were less likely to achieve glycemic control (P<0.001). Glycemic control was highly associated with blood glucose measurement in the past month; patients who took these measurements were less likely to have their glucose levels controlled.

*Patient-related aspects of healthcare quality*. As presented in Table III, 479 out of 688 patients (70%) adhered to their specified appointments. Approximately 61% of the patients owned a glucose testing device. The proportion of patients who missed medications was 28.6%, while 70.8% rarely missed taking their medications during travel, and 6.6% always stopped taking their medications when they felt better. Approximately 10% of the patients performed light physical activity for <1 h/week, while 61.3% performed light physical activity for 1-9 h/week. Of the patients, >15% spent <1 h/week performing moderate-intensity physical activity. Approximately 65% of the patients did not engage in high-intensity physical activity. Approximately 3% of the patients spent <1 h/week performing any form of physical activity. In total, >38% of the patients reported that places to perform physical activity were available. As regards diet, ~51% of the patients were on a specific diet for managing diabetes during the week prior to data collection. In addition, >32% of the patients had been living with diabetes for <4 years, while 42.2% had been living with diabetes for >9 years.

Adherence to appointments was significantly associated with glycemic control; patients who adhered to specified appointments had a lower glycemic control (P=0.018), as shown in Table III. Patients who stopped taking medications after feeling healthy had better glycemic control (P=0.006). Glycemic control was found to be significantly associated with moderate-intensity physical activity, and longer hours of moderate-intensity physical activity were associated with better glycemic control (P=0.039). However, disease duration was inversely associated with glycemic control (P<0.001).

### Factors independently related to glycemic control (HbA1c level <7%)

Binary logistic regression analysis was performed to determine the factors independently related to glycemic control (HbA1c level <7%) after controlling for potential confounders. In the first step, all variables with significant or close-to-significant associations in bivariate analyses were included in the model. Subsequently, the regression was run multiple times based on excluding the least significant variables to finally end up with the best fitting model, which included the least number of variables that best explained the variability in glycemic control. As shown in Table IV, the diabetes duration was significantly associated with glycemic control. Living with diabetes for longer durations was associated with poor glycemic control. Patients who had been living with diabetes for <4 years were 3.95-fold more likely to have controlled blood glucose levels than those who were diagnosed for >9 years, after controlling for other variables in the model [adjusted odds ratio (OR), 3.95; 95% confidence interval (CI), 2.54-6.14; P<0.001]. Being on a special diet for diabetes was found to be significantly associated with glycemic control. Those who followed a specific diet for diabetes had a 58% higher likelihood of glycemic control than those who did not follow a diet, after controlling for other variables in the model (adjusted OR, 1.58; 95% CI, 1.08-2.30; P=0.018). The number of annual visits was also significantly associated with glycemic control. Patients who visited the physician 1-4 times/year were 2.36-fold more likely to achieve glycemic control (HbA1c level <7%) than those who visited the physician >10 times/year, after controlling for other variables in the model (adjusted OR, 2.36; 95% CI, 1.57-3.54; P<0.001).

The cessation of medication after feeling better was associated with better glycemic control after controlling for other variables in the model. Patients who always stopped taking their medications after feeling better were twice as likely to have glycemic control compared with those who rarely quit their medications after controlling for other variables in the model (adjusted OR, 2.11; 95% CI, 1.04-4.29; P=0.038).

Multivariate analysis was also performed to identify the factors related to glycemic control (HbA1c level <7.5%). Variables related to glycemic control with a target HbA1c level of <7.5% were the same as those related to glycemic control with a target HbA1c level of <7%, except for the variable ‘medication cessation after feeling better’, which was not related to a target HbA1c level of <7.5%.

### Factors independently related to hospital admission owing to diabetes during the past year

A similar analysis of glycemic control was performed to identify the variables associated with hospital admission owing to diabetes after controlling for other potential confounders. As presented in Table V, the number of years that patients lived with diabetes was significantly associated with hospital admission owing to diabetes. Patients who were living with diabetes for >9 years were 2.11-fold more likely to be admitted than those who were living with diabetes for <4 years, after controlling for other variables in the model (adjusted OR, 2.11; 95% CI, 1.18-3.78; P=0.012). The number of years of education was significantly associated with hospital admissions owing to diabetes. Those who were illiterate were 4.06-fold more likely to be admitted to the hospital than those who received >12 years of education, after controlling for other variables in the model (adjusted OR, 4.06; 95% CI, 1.85-8.89; P<0.001). Measuring cholesterol levels in the past was significantly related to hospital admissions owing to diabetes. Patients who had their cholesterol levels measured were twice as likely to be admitted owing to diabetes compared with those who did not have their cholesterol levels measured, after controlling for other variables in the model (adjusted OR, 2.01; 95% CI, 1.14-3.54; P=0.015).

## Discussion

Assessing healthcare delivery for diabetes is complex and is related to the adherence to international recommendations, the achievement of treatment goals, such as glycemic control, and the prevention of possible complications. The present study demonstrated the level of healthcare services provided to patients with type 2 diabetes and patients' health-related behaviors, and this was the first study in Jordan to describe the quality of healthcare delivered to patients with type 2 diabetes. Therefore, the findings presented herein may be used to guide diabetes clinical planning and management, given that the study also evaluated laboratory and anthropometric data and did not only rely on self-reported information.

### Provider-related aspects of healthcare quality

One important finding of the present study was that health insurance covered a high proportion (85.8%) of patients with diabetes who were aged ≥25 years. The insurance coverage in the present study did not reveal a significant difference between glycemic control with a target HbA1c level of <7% and >7%. This was not consistent with findings of studies conducted in the USA. The National Health and Nutrition Examination Survey found that uninsured individuals were more likely to have HbA1c levels of >9% than insured individuals (9). A possible explanation of this finding is that the uninsured patients in the present study had good access to medical care, in addition to the highly affordable costs of the Ministry of Health services and other insurance channels, such as ‘exemptions’ and ‘coverage of the poor’, which cover medical costs in Jordan. Thus, contrary to the USA, where patients bear high costs for medical services if they are not insured, patients with diabetes in Jordan can access medical care at no or very low costs, irrespective of their insurance status.

The number of annual visits in the present study ranged from 0 among 6.6% of the patients to >10 visits among 48.5% of the patients. The mean annual number of visits was 7.3. The ADA recommends at least 2-4 annual visits (10). The number of annual visits in the present study exhibited a significant inverse association with glycemic control (HbA1c level <7%) in bivariate and multivariate analyses. Patients who visited the health facility 1-4 times/year were 2.36-fold more likely to have glycemic control than those who visited the health facility >10 times/year, after controlling for potential confounders (P<0.001). This may be attributed to uncontrolled hyperglycemia that leads to an increased number of visits or the quality of these visits not meeting the patient's therapeutic needs (11,12).

The present study also found that 81.6% of the patients reported easy access to health facilities. This was expected, as medical centers are spread across Jordan and the majority of patients do not need to drive a long distance to access medical services; on average, the distance to a health center is <30 min. Reporting easy access to a health facility in the present study was not significantly different between patients with and without glycemic control, while a study in Pennsylvania reported a significant difference in glycemic control in relation to the distance taken to drive to the health facility, pointing to travel burden as a factor influencing glycemic control (13).

According to the ADA, foot examinations should be performed annually, while eye examinations must be performed when diabetes is diagnosed and then every 1-2 years (10). The present study revealed that the proportion of patients who had their feet and eyes examined was relatively low (24.9 and 54.6%, respectively). This may be explained by the poor referral practices of care providers or poor patient compliance. The data revealed a fair adherence to appointments (~70%), suggesting that both poor referral practices and poor compliance may be responsible for the low foot and eye examinations observed in our study. Poor referral and the compliance of patients could be related to the large number of patients attending primary healthcare clinics indicating a short time of visits that is solely given for treatment.

Levels of glycemic control (i.e., HbA1c) are commonly used as a measure of diabetes care quality and achieving a high glycemic control could serve as a good indicator for a satisfactory level of diabetes care. The present study revealed that the proportions of patients achieving glycemic control with target HbA1c levels of <7 and <7.5% were 33.0 and 41.5%, respectively. Compared with results of a study evaluating glycemic control in eight European countries, these proportions were low; a European study revealed that 53.6% of the patients achieved glycemic control with target HbA1c levels of <7% (14). According to the data obtained from the Centers for Disease Control and Prevention, the percentage of adults in the United States with diabetes achieving an HbA1c target of <7% has been constant from 2003 to 2010, remaining at >50% (15). However, glycemic control in Jordan, as revealed in the present study, was better than that reported in a Brazilian study (20%) based on the same target HbA1c level (16). The prevalence of glycemic control (HbA1c level <7%) in Jordan was also better than that in other countries, such as Bangladesh, where the proportion of patients with glycemic control did not exceed 18% based on the same HbA1c cutoff value (17).

An important aspect of diabetes care assessment is the performance of certain recommended screening tests. These include tests for measuring blood glucose, blood pressure and cholesterol levels, and the proportions of patients who underwent these tests during the past month were 73.6, 59.3 and 23.8%, respectively. Having blood glucose levels measured during the past month was found to be significantly associated with poor glycemic control. Patients who performed these tests were less likely to achieve glycemic control (HbA1c level <7%). This finding indicated that these patients had their blood glucose levels measured as they experienced rises in their levels, and repeated testing is required due to uncontrolled hyperglycemia. Furthermore, patients who underwent cholesterol tests were twice as likely to be admitted to the hospital compared with those who did not have their cholesterol levels measured, after controlling for other variables in the model (P=0.015), which was expected as blood cholesterol tests, among other tests, are performed routinely for hospitalized patients with diabetes. Team support was another healthcare quality indicator significantly related to hospital admission owing to diabetes. Approximately 3 in 4 (78.7%) patients reported that they received good health team support, which is a key psychological support factor for patients with diabetes.

The present study also demonstrated the suboptimal proportions of patients receiving advice regarding diet, going to the gym, and losing weight (87.5, 75.2 and 72.1%, respectively) from their doctors. The majority of physicians are not trained in nutrition intervention, which is a barrier to advising patients. Moreover, numerous clinics are overwhelmed with a high number of patients, who receive their regular consultations for short durations and leave the clinic with only a prescription of medications. Multiple studies have shown that providing patients with advice and education is highly effective in controlling hyperglycemia (18,19); therefore, it is critical that all patients receive advice and education about diet, weight loss and physical activity for diabetes management (the target should be close to 100%).

### Patient-related aspects of healthcare quality

The importance of physical activity and following a special diet is demonstrated in the findings of the present study. First, in the bivariate analysis, performing moderate-intensity physical activity was significantly associated with better glycemic control (HbA1c level <7%). The likelihood of glycemic control increased with an increase in the hours of moderate-intensity physical activity (P=0.039). Second, in the multivariate analysis, following a specific diet for diabetes was associated with a 58% higher likelihood of glycemic control after controlling for other variables in the model. The findings concerning the positive effects of physical activity were consistent with those of previous studies (20,21). According to the ADA, adults with type 1 and type 2 diabetes should engage in 150 min or more of aerobic activity per week, spread over at least 3 days/week. Shorter durations (minimum 75 min/week) of training may be sufficient for younger and more physically fit individuals (22).

As was expected, the number of years since diabetes diagnosis was significantly associated with poor glycemic control. Patients diagnosed <4 years earlier were 3.95-fold more likely to have controlled blood glucose levels than those who were diagnosed >9 years, after controlling for other variables in the regression model (P<0.001), which was consistent with national and international studies (13,23-25). In line with this result, a study conducted in Hawaii in the USA reported that patients who had diabetes for >10 years were more likely to have poor glycemic control than those who had diabetes for 3 years (26). This may be owing to the progressive impairment of insulin secretion by β-cells and an increase in insulin resistance over time. Another expected finding was that the number of years since diabetes diagnosis was significantly associated with hospital admission owing to diabetes. Patients who were diagnosed >9 years earlier were 2.11 times more likely to be hospitalized owing to diabetes than those who were diagnosed later (<4 years), after controlling for variables in the model (P=0.012).

Contrary to what was expected, patients who always stopped taking their medications after feeling better were twice as likely to have controlled blood glucose levels compared with those who rarely quit their medications, after adjusting for the number of years with diabetes, whether a special diet was followed, and the number of annual visits (adjusted OR, 2.11; P=0.038). This finding was in contrast to those reported in other studies. For example, a retrospective cohort study conducted in Singapore revealed that the HbA1c level of poorly adherent patients increased by 0.4% over the 2-year follow-up period, and they were more likely to be hospitalized or have emergency room visits than those who were fully adherent to their medications (27). Another study conducted in northwest Oregon and southwest Washington in the USA reported that a higher adherence to oral hypoglycemic medications was independently associated with a lower likelihood of poor glycemic control among adults with type 2 diabetes (28). However, a study conducted in Saudi Arabia found no association between glycemic control and adherence to oral antidiabetic drugs (29). In the current study, better glycemic control in patients who quit their medications after feeling better may be owing to mild diabetes. Moreover, ~87% of the patients in this group did not require hospital admission, 80% did not visit the emergency room owing to diabetes, and ~89% of them performed moderate-intensity physical activity for >1 h/week; these factors support the hypothesis that these patients had a mild form of diabetes.

Another important aspect of medical information for patients with diabetes is their family history. It was found that 70.6% of the patients had a family history of diabetes, which was consistent with the literature (30). Family history is a strong risk factor for type 2 diabetes, as it was found to be present in the majority of patients with type 2 diabetes. However, the incidence of diabetes is complex and multifactorial. In addition, although the incidence of diabetes and its related risk factors are important to evaluate, disease management is key in reducing the risk of possible complications that usually manifest in patients with poorly controlled diabetes.

Diabetes rates are increasing in Jordan and healthcare providers who are well trained to manage cases of diabetes do not match the rising needs of care, which develop a serious challenge to the healthcare system and providers. In addition, patients' education and awareness campaign are not integral parts of diabetes management in Jordan, which therefore, increase the burden on diabetologists and primary care doctors to take the role of educating patients. Yet, educating patients in clinics is rarely occurring due to the high volume of patients attending public clinics.

In conclusion, the present study provides a number of indicators for the quality of care for diabetes in Jordan. Although many of these were relatively satisfactory, there is scope to improve some factors. Glycemic control (HbA1c level <7%) was independently and inversely associated with the number of years with diabetes and number of annual visits. Following a specific diet for managing diabetes and medication cessation after feeling better were independently associated with higher odds of glycemic control (HbA1c level <7%). A number of patients in Jordan tend to not regularly visit health facilities unless there is an emergency, their disease is not controlled, or they become ill. However, patients who had been living with diabetes for long may have developed complications that needed attention, and those diagnosed in the past few years had lower levels of awareness about the importance of regular visits. Education is a major contributor to the quality of care provided to patients with diabetes. Hence, it is imperative to educate all patients with diabetes, including those who are newly diagnosed and at risk, about the importance of regularly visiting health facilities, and the educational material should include information about diabetes treatment, management, and possible complications.

## Figures and Tables

**Table I:** Associations between glycemic control (HbA1c level <7%) and the sociodemographic characteristics of patients with diabetes in Jordan (2017).

	Glycemic control (HbA1c level <7%)			
Variable	Yes, n (%)	No, n (%)	Total	P-value
Sex				0.003
Male	88 (27.2)	235 (72.8)	323	
Female	161 (37.4)	270 (62.6)	431	
Age (years)				0.002
25-39	27 (51.9)	25 (48.1)	52	
40-49	62 (38.5)	99 (61.5)	161	
50-59	87 (32.1)	184 (67.9)	271	
≥60	73 (27.0)	197 (73.0)	270	
Marital status				0.493
Currently married	205 (32.6)	424 (67.4)	629	
Currently unmarried^a^	44 (35.8)	79 (64.2)	123	
Years of education				0.007
Illiterate	19 (22.9)	64 (77.1)	83	
1-12	151 (31.3)	332 (68.7)	483	
>12	70 (41.4)	99 (58.6)	169	
Nationality				0.651
Jordanian	233 (32.6)	482 (67.4)	715	
Others	12 (36.4)	21 (63.6)	33	
Family history of diabetes				0.364
Yes	182 (34.0)	353 (66.0)	535	
No	67 (30.6)	152 (69.4)	219	
Body mass index^b^				0.285
Normal	20 (32.3)	42 (67.7)	62	
Overweight	72 (29.4)	173 (70.6)	245	
Obese	155 (35.3)	284 (64.7)	439	

^a^Currently unmarried comprised those who were single, divorced, separated and widowed.

^b^Data of underweight participants (n=4) were treated as missing. The variables marital status, nationality, family history and body mass index were not significantly associated with glycemic control (HbA1c level <7%). Data were analysed using the Chi-squared test of independence.

**Table II:** Associations between glycemic control (HbA1c level <7%) in patients with type 2 diabetes and healthcare provider aspects in Jordan (2017).

	Glycemic control (HbA1c <7%)			
Variable	Yes, n (%)	No, n (%)	Total	P-value
Healthcare provider				0.260
Ministry of Health	135 (33.2)	272 (66.8)	407	
Military Services	60 (29.0)	147 (71.0)	207	
University + private	34 (41.0)	49 (59.0)	83	
Others	19 (35.2)	35 (64.8)	54	
Insurance				0.949
Insured	213 (33.0)	432 (67.0)	645	
Uninsured	36 (33.3)	72 (66.7)	108	
Number of annual visits				<0.001
None	17 (38.6)	27 (61.4)	44	
1-4	99 (38.1)	161 (61.9)	260	
5-9	18 (39.1)	28 (60.9)	46	
>10	65 (19.8)	264 (80.2)	329	
Easy access to health facilities				0.063
Yes	159 (28.4)	401 (71.6)	560	
No	46 (36.8)	79 (63.2)	125	
Has your blood glucose been measured in the past month?				0.002
Yes	164 (29.7)	388 (70.3)	552	
No	83 (41.9)	115 (58.1)	198	
Has your blood glucose ever been measured in the past?				0.009
Yes	229 (31.9)	488 (68.1)	717	
No	16 (55.2)	13 (44.8)	29	
Has your blood pressure been measured in the past month?				0.917
Yes	148 (33.3)	297 (66.7)	445	
No	100 (32.9)	204 (67.1)	304	
Has your cholesterol level been measured in the past month?				0.415
Yes	54 (30.3)	124 (69.7)	178	
No	192 (33.6)	379 (66.4)	571	
Has your cholesterol level ever been measured in the past?				0.341
Yes	158 (31.7)	340 (68.3)	498	
No	89 (35.2)	164 (64.8)	253	
Have your feet ever been examined?				0.159
Yes	44 (25.7)	127 (74.3)	171	
No	164 (31.4)	358 (68.6)	522	
Have your eyes ever been examined?				0.740
Yes	112 (29.6)	266 (70.4)	378	
No	97 (30.8)	218 (69.2)	315	
Health team support as stated by the patient				0.074
Good	151 (28.0)	388 (72.0)	539	
Poor	52 (35.6)	94 (64.4)	146	
Does the medical team explain the disease to the patient?				0.118
Never	35 (27.3)	93 (72.7)	128	
Sometimes	85 (34.3)	163 (65.7)	248	
Always	81 (26.6)	224 (73.4)	305	
Do medical teams answer questions regarding diabetes?				0.293
Never	33 (30.8)	74 (69.2)	107	
Sometimes	72 (33.2)	145 (66.8)	217	
Always	96 (27.1)	258 (72.9)	354	
Do medical teams listen to the patient?				0.183
Never	38 (32.5)	79 (67.5)	117	
Sometimes	77 (32.8)	158 (67.2)	235	
Always	86 (26.2)	242 (73.8)	328	
Doctor advised patients to follow a diet				0.892
Yes	178 (29.9)	418 (70.1)	596	
No	26 (30.6)	59 (69.4)	85	
Doctor advised patients to go to the gym				0.725
Yes	153 (30.4)	351 (69.6)	504	
No	48 (28.9)	118 (71.1)	166	
Doctor advised patients to lose weight				0.371
Yes	150 (31.1)	332 (68.9)	482	
No	51 (27.6)	134 (72.4)	185	
Emergency room visit during past year owing to diabetes				0.417
Yes	48 (27.6)	126 (72.4)	174	
No	161 (30.8)	361 (69.2)	522	
Hospital admission during past year owing to diabetes				0.195
Yes	22 (24.2)	69 (75.8)	91	
No	186 (30.8)	417 (69.2)	603	

Data were analysed using the Chi-squared test of independence.

**Table III:** Associations between glycemic control (HbA1c level <7) in patients with type 2 diabetes and patient-related aspects of healthcare in Jordan (2017).

	Glycemic control HbA1c level <7%			
Variable	Yes, n (%)	No, n (%)	Total	P-value
Adherence to appointments				0.018
Yes	134 (28.0)	345 (72.0)	479	
Sometimes	42 (30.7)	95 (69.3)	137	
No	32 (44.4)	40 (55.6)	72	
Having a glucose testing device				0.094
Yes	116 (27.6)	304 (72.4)	420	
No	92 (33.6)	182 (66.4)	274	
Missed taking medications				0.999
Always	13 (29.5)	31 (70.5)	44	
Sometimes	58 (29.7)	137 (70.3)	195	
Rarely	133 (29.8)	313 (70.2)	446	
Missed taking medications during travel				0.601
Always	18 (32.7)	37 (67.3)	55	
Sometimes	46 (32.2)	97 (67.8)	143	
Rarely	138 (28.5)	347 (71.5)	485	
Medication cessation after feeling better				0.006
Always	21 (47.7)	23 (52.3)	44	
Sometimes	39 (34.2)	75 (65.8)	114	
Rarely	139 (26.7)	381 (73.3)	520	
Availability of places for physical activity				0.412
Yes	85 (31.8)	182 (68.2)	267	
No	124 (28.9)	305 (71.1)	429	
Light physical activity (hours/week)				0.977
<1	21 (30.4)	48 (69.6)	69	
1-9	145 (32.7)	298 (67.3)	443	
10-19	31 (31.3)	68 (68.7)	99	
≥20	36 (32.7)	74 (67.3)	110	
Moderate-intensity physical activity (hours/week)				0.039
<1	27 (23.7)	87 (76.3)	114	
1-9	109 (31.7)	235 (68.3)	344	
10-19	38 (36.5)	66 (63.5)	104	
≥20	65 (39.4)	100 (60.6)	165	
High-intensity physical activity (hours/week)				0.324
0	145 (30.9)	324 (69.1)	469	
0.5-3.0	54 (25.3)	99 (64.7)	153	
>4	38 (37.6)	63 (62.4)	101	
Overall physical activity for < 1 hour/week				0.210
Yes	4 (20.0)	16 (80.0)	20	
No	238 (33.4)	475 (66.6)	713	
Followed a special diet for diabetes during the last week				0.069
Yes	116 (33.1)	234 (66.9)	350	
No	90 (26.8)	246 (73.2)	336	
Number of years living with diabetes				<0.001
<4	107 (47.8)	117 (52.2)	224	
4-9	44 (25.6)	128 (74.4)	172	
>9	47 (16.4)	239 (83.6)	286	

Data were analysed using the Chi-squared test of independence.

**Table IV:** Results of the multivariate logistic regression analysis to determine factors associated with glycemic control (HbA1c level <7%) among patients with type 2 diabetes in Jordan (2017).

		95% CI		
Variable	Adjusted OR	Lower limit	Upper limit	P-value
Number of years with diabetes				<0.001
<4	3.95	2.54	6.14	<0.001
4-9	1.50	0.91	2.46	0.114
>9	1.00			
Followed a special diet for managing diabetes during the last week				0.018
Yes	1.58			
No	1.00	1.08	2.30	
Number of annual visits				<0.001
0	1.93	0.91	4.09	0.088
1-4	2.36	1.57	3.54	<0.001
5-9	2.24	1.10	4.53	0.026
>10	1.00			
Medication cessation after feeling better				0.100
Always	2.11	1.04	4.29	0.038
Sometimes	1.23	0.76	2.00	0.402
Rarely	1.00			

The reference group was the one with the least risk. Multivariate logistic regression analysis was used for data analysis. OR, odds ration; CI, confidence interval.

**Table V:** Variables independently related to hospital admission owing to diabetes during the past year of patients with type 2 diabetes in Jordan (2017).

		95% CI		
Variable	Adjusted OR	Lower limit	Upper limit	P-value
Number of years of education				0.001
0	4.06	1.85	8.89	<0.001
1-12	1.57	0.82	2.99	0.171
>12	1.00			
Number of years living with diabetes				0.041
<4	1.00			
4-9	1.54	0.79	3.03	0.206
>9	2.11	1.18	3.78	0.012
Has your cholesterol level been measured in the past?				0.015
Yes	2.01			
No	1.00	1.14	3.54	

The reference group was the one with the least risk. Multivariate logistic regression analysis was used for data analysis. OR, odds ration; CI, confidence interval.

## Data Availability

The datasets generated and/or analyzed during the current study are available from the corresponding author on reasonable request.
